# Enlarged Interlayer Spacing of Marigold-Shaped 1T-MoS_2_ with Sulfur Vacancies via Oxygen-Assisted Phosphorus Embedding for Rechargeable Zinc-Ion Batteries

**DOI:** 10.3390/nano13071185

**Published:** 2023-03-27

**Authors:** Qinhu Xu, Xinyu Li, Luchen Wu, Zhen Zhang, Yong Chen, Ling Liu, Yong Cheng

**Affiliations:** College of Science & Ministry-Province Jointly-Constructed Cultivation Base for State Key Laboratory of Processing for Mom-Ferrous Metal and Featured Materials & Key Lab. of Nonferrous Materials and New Processing Technology & Network and Information Center, Guilin University of Technology, Guilin 541004, China

**Keywords:** interlayer-expanded MoS_2_, sulfur vacancies, phosphorus embedding, aqueous Zn-ion batteries

## Abstract

Structural unsteadiness and sluggish diffusion of divalent zinc cations in cathodes during cycling severely limit further applications of MoS_2_ for rechargeable aqueous zinc-ion batteries (ZIBs). To circumvent these hurdles, herein, phosphorus (P) atom embedded three-dimensional marigold-shaped 1T MoS_2_ structures combined with the design of S vacancies (Sv) are synthesized via the oxygen-assisted solvent heat method. The oxygen-assisted method is utilized to aid the P-embedding into the MoS_2_ crystal, which can expand the interlayer spacing of P-MoS_2_ and strengthen Zn^2+^ intercalation/deintercalation. Meanwhile, the three-dimensional marigold-shaped structure with 1T phase retains the internal free space, can adapt to the volume change during charge and discharge, and improve the overall conductivity. Moreover, Sv is not only conducive to the formation of rich active sites to diffuse electrons and Zn^2+^ but also improves the storage capacity of Zn^2+^. The electrochemical results show that P-MoS_2_ can reach a high specific capacity of 249 mAh g^−1^ at 0.1 A g^−1^. The capacity remains at 102 mAh g^−1^ after 3260 cycles at a current of 0.5 A g^−1^, showing excellent electrochemical performance for Zn^2+^ ion storage. This research provides a more efficient method of P atom embedded MoS_2_-based electrodes and will heighten our comprehension of developing cathodes for the ZIBs.

## 1. Introduction

Due to the distinct characteristics of zinc metal, such as high theoretical capacity (820 mAh g^−1^), low redox potential (−0.76 V concerning standard hydrogen electrodes), nontoxicity, low cost, and intrinsic safety, as well as high reversibility in aqueous electrolytes, ZIBs hold promising potential for large-scale energy storage [1,2,3,4]. However, Zn^2+^ in aqueous solution is hard to intercalate between layers because it exists as hydrated zinc ions ([Zn(H_2_O)_6_]^2+^) with a size of 0.404 nm~0.43 nm, making ([Zn(H_2_O)_6_]^2+^) intercalation and deintercalation put forward higher requirements on the interlayer spacing and other properties of the cathode material [5,6,7]. In addition, divalent Zn^2+^ usually exhibits strong electrostatic interactions with the host lattice, inhibiting the diffusion process of Zn^2+^, resulting in few cathode materials available for ZIBs, which are mainly confined to manganese-based materials, vanadium-based materials, and Prussian blue [8,9,10]. Therefore, the sluggish intercalation dynamics of divalent Zn^2+^ enable the search for appropriate cathode materials to be very important for the development and application of ZIBs.

Molybdenum disulfide (MoS_2_) is a typical layered transition metal sulfide, and the covalently bonded S-Mo-S layers are stacked together by weak van der Waals forces, which is conducive to the intercalation of foreign guests [11,12,13,14]. Unfortunately, due to the strong electrostatic interaction between inserted Zn^2+^ with a large hydrated radius (4.3 Å) and host structures, Zn^2+^ is challenging to intercalate/deintercalate from the host frame, greatly affecting the reversible capability and rate characteristic of MoS_2_ as cathodes arousing from its smaller layer spacing and the large inert base with fewer active sites [15,16,17,18]. Moreover, MoS_2_ also has problems such as poor conductivity, poor hydrophilicity, agglomeration, and volume expansion, which limits the application of MoS_2_ in ZIBs. Therefore, some pioneering efforts, including introducing phase transition engineering and tailoring nanostructures, have been made to improve the Zn^2+^ storage capability of MoS_2_ cathodes [19,20,21,22]. One available strategy to resolve such challenges is to intercalate foreign elements into MoS_2_ to enlarge layer spacing [15,23,24]. Experimental and calculation results indicate that P dopants could not only modify the surface electronic state of MoS_2_ and increase its inherent conductivity but also lead to MoS_2_ expansion and induce a partial phase transition of MoS_2_ from hydrophobic (2H phase) to hydrophilic (1T phase); the 1T-MoS_2_ has lower Zn diffusion energy barriers [25]. However, due to the spontaneous formation of MoP, resulting in P atom doping is inherently difficult and has lower P content [26,27]. Despite substantial achievements having been made, P-embedding 1T-MoS_2_ to achieve extended layer spacing for enhancing the ability of Zn^2+^ intercalates/deintercalates is very necessary, thus requiring a facile method.

Additionally, vacancy engineering has been in the spotlight as a feasible strategy to enhance the zinc ions storage capability considerably. Especially based on previous reports [28], sulfur vacancies can provide abundant active sites as additional ion storage sites for Zn^2+^ as well as offer a fast electrochemical response. For example, Hu et al. [29] developed a few-layered MoS_2_ anchored on an N-doped carbon flower with Sv as anode material for sodium-ion batteries, exhibiting excellent performance. Wang et al. [30] improved the performance of hydrogen evolution reaction (HER) by regulating the S-vacancy distribution and concentration in MoS_2_. Xu et al. reported that the preparation of defect-rich MoS_2_ accelerated the diffusion kinetics of Zn^2+^ to the active center [31]. Therefore, the rational S vacancy modulation strategy is essential to improve the reaction kinetics of Zn^2+^ for realizing its application in ZIBs.

Herein, an oxygen-assisted(O) strategy is used to aid the P-embedding into the MoS_2_ crystal; the MoS_2_ layer spacing is expanded, and the marigold-shaped 1T-MoS_2_ nanosheets with rich S vacancies are prepared. In this process, oxygen played a key role in aiding the successful embedding of P into the lattice of 1T-MoS_2_. The intercalation of P in MoS_2_ achieves the desired effect, significantly enlarging MoS_2_ interlayers (from 0.62 to 0.98 nm) and enhancing hydrophilicity. In addition, these S vacancies defects as active sites can make it easier for Zn^2+^ adsorption and desorption. The synergistic effect of P-embedding induced and extended the marigold-shaped 1T-MoS_2_ layer spacing and caused the Sv to form an active center in the basal plane of MoS_2_, effectively reducing the Zn^2+^ diffusion resistance. It provides an easier channel for the insertion of [Zn(H_2_O)_6_]^2+^, resulting in rapid reaction kinetics. As expected, the P-MoS_2_ electrode achieves a remarkably high capability of 249 mAh g^−1^ at 0.1 A g^−1^, which is five times higher than the specific capacity of pristine MoS_2_. In comparison to the pristine P-MoS_2_ counterpart with minimal capacity delivery, P-MoS_2_ can achieve a high reversible capacity of 105 mAh g^−1^ at 0.5 A g^−1^ with 3260 cycles and excellent capacity retention of 70 mAh g ^−1^ at 1 A g^−1^. This study will provide more efficient avenues for investigating more electrode materials with poor intercalation kinetics in ZIBs.

## 2. Methods

### 2.1. Experimental Section

Synthesis of P-MoS_2_: In a representative procedure, 1 mmol (1236 mg) ammonium molybdate tetrahydrate ((NH_4_)_6_Mo_7_O_24_·4H_2_O, AR) and 30 mmol (2284 mg) thiourea (CH_4_N_2_S, AR) were added into 35 mL deionized water on the basis of a beforehand report with slight adjustment [32]. After being stirred for 30 min, an amount of sodium hypophosphite monohydrate (NaH_2_PO_2_·4H_2_O, AR, 400 mg) was dissolved into a mixed solution with magnetic stirring for 2 h. Then, the precursor solution was heated to 180 °C for 24 h in a 50 mL Teflon-lined stainless steel autoclave. After cooling the reaction system to air temperature, which was collected by centrifugation, cleaned multiple times with water, and then dried at 80 °C. As a contrast, pristine MoS_2_ was also prepared without NaH_2_PO_2_·4H_2_O following a similar synthetic route.

### 2.2. Material Characterizations

The morphology of materials was evaluated with a scanning electron microscope (SEM, Hitachi S-4800, Tokyo, Japan) and a transmission electron microscope (TEM, Tecnai G2 20 TWIN, FEI, Hillsboro, OR, USA). An energy dispersive spectrometer (EDS S-00123, USA) connected to the SEM was used to study the elemental composition and elemental analyses of the composites. At −196 °C, N_2_ adsorption/desorption isotherms were measured with an automated SSA and porosity analyzer (asap2460). Brunauer–Emmett–Teller (BET) and Barrett–Joyner–Halenda (BJH) adsorption techniques were used to determine the SSA and mesopore size distribution of each sample. All samples’ crystal phases and compositions were determined using X-ray diffraction (XRD, MiniFlex-600, Rigaku, Tokyo, Japan) and X-ray photoelectron spectroscopy (XPS, Thermo Scientific K-Alpha, Waltham, MA, USA). Raman spectra at 532 nm were measured with a Horiba Scientific LabRAM HR Evolution Raman spectrometer. The TGA/DTA was tested by thermogravimetric analysis equipment (SDT Q600, USA) from room temperature to 750 °C at a heating rate of 10 °C/min. A hydrophilicity test was measured using a contact angle test device (Dataphysics OCA20, Filderstadt, Germany).

### 2.3. Electrochemical Characterization

Zn served as the anode, glass fiber membrane served as diaphragms, and 3 M Zn(CF_3_SO_3_)_2_ served as the electrolyte in CR2016-type coin cells used to investigate the Zn^2+^ storage properties of P-MoS_2_. To manufacture the working electrode, P-MoS_2_ (70 weight percent), super p carbon (20 weight percent), and polyvinylidene fluoride (10 weight percent) were thoroughly blended in *N*-methyl-2-pyrrolidone for 15 min. Finally, the aforementioned slurry was distributed over a clean stainless steel mesh and dried for 24 h at 60 °C. On a Neware battery tester (CT4008), galvanostatic charge/discharge measurements and galvanostatic intermittent titration technique (GITT) experiments were carried out continuously between 0.25 and 1.25 V (vs. Zn/Zn^2+^). An electrochemical workstation was used for the cyclic voltammetry (CV) and electrochemical impedance spectroscopy (EIS) experiments (CHI-660D). When the batteries were completely charged, impedance measurements were taken (zinc extraction). At room temperature, all tests were conducteddw.

## 3. Results and Discussion

### 3.1. Composition and Structure

**Figure 1:**
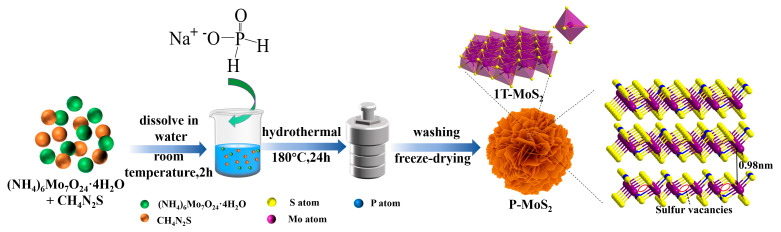
Schematic illustration of synthesis P-MoS_2_.

**Figure 2:**
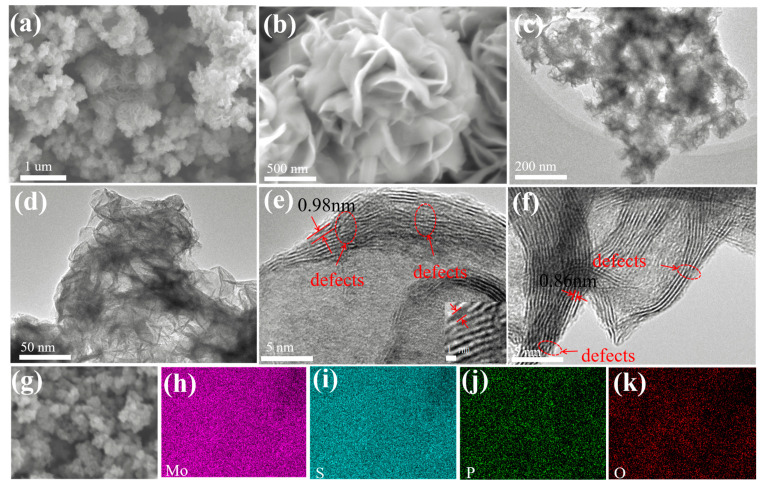
(**a**,**b**) SEM; (**c**,**d**) TEM; (**e**,**f**) HRTEM; (**g**–**k**) EDS of P-MoS_2_.

Figure 1 depicts the synthesis process of P-MoS_2_ and pristine MoS_2_. The phosphorus embedded three-dimensional marigold 1T MoS_2_ was obtained by controlling the crystallization process. In a nutshell, an oxygen-rich atmosphere plays a vital role in decreasing the formation energy of P-embedding in MoS_2_ [26]. The reaction process becomes more inadequate as the synthesis temperature decreases, resulting in the leftover oxygen inherited from the molybdate precursor, thus realizing the oxygen-rich atmosphere [32]. Additionally, most of the Mo^4+^ ions form ionic bonds with S^2−^, which are self-assembled in the form of nanocrystals connected into three-dimensional marigold-shaped structures. Excess thiourea could be adsorbed on the surfaces of initial nanocrystallites and impede the formation of orientated crystals, which results in abundant Sv being produced amid the flat structure [33]. The SEM and TEM were utilized to explore the morphology evolution of as-prepared P-MoS_2_ further. As shown in Figure 2a,b, the SEM of P-MoS_2_ displays the magnificent marigold structure with an internal diameter of almost 1 µm, which was self-assembled from curved thickness nanosheets with transverse dimensions of 400–500 nm. In comparison, pristine MoS_2_ has a rather formidable nanosheet microstructure, which is much larger in transverse size (diameter almost 4–5 µm) and displays severe aggregation in the absence of a well-tuned morphology (Figure S1). The relatively smaller size of the beautiful marigold structure should help to shorten the Zn^2+^ diffusion duration. The uneven nanostructure of P-MoS_2_ with evident ripples and bending wrinkles is depicted in TEM images (Figure 2c,d), in which nanosheets are haphazardly joined and formed into three-dimensional structures. The TEM images in Figure 1e,f reveal a distinct lattice structure with some disorder and abundance of Sv (Figure S2), resulting in an incomplete lattice that may accommodate a host of unsaturated S atoms as active sites. Furthermore, the TEM images reveal that dozens of pristine MoS_2_ layers are heavily stacked together with an interlayer spacing of 0.62 nm (Figure S3), whereas the stacking of the P-MoS_2_ layers is significantly relieved with the interlayer distance strikingly expanding to 0.98 (Figure S4) and 0.86 nm. The results show that P-embedding can enlarge the layer spacing of P-MoS_2_, which is advantageous to strengthen Zn^2+^ intercalates/deintercalates. Figure 2h–k displays the mapping images of P-MoS_2_, which expressly demonstrate that Mo, O, S, and P elements are distributed uniformly. Meanwhile, Figure S5 shows the elemental content of P-MoS_2_.

**Figure 3:**
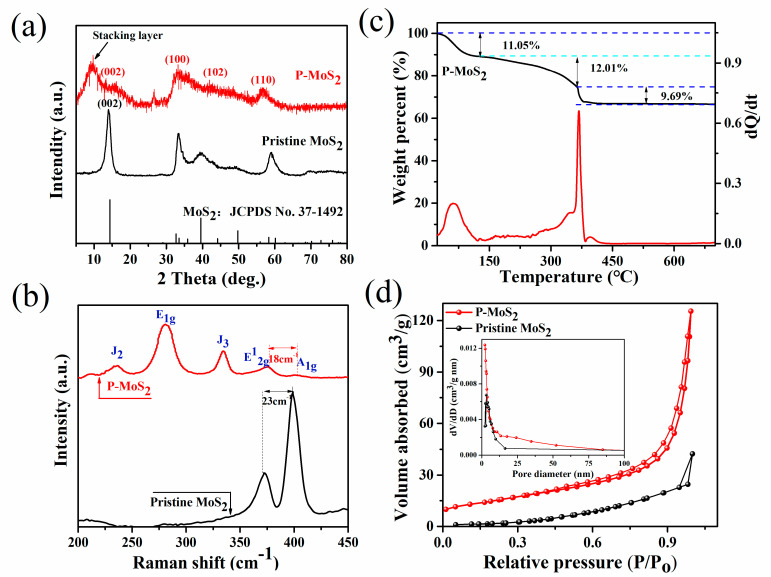
(**a**) XRD patterns of P-MoS_2_ and pristine P-MoS_2_. (**b**) Raman spectra of P-MoS_2_ and pristine MoS_2._ (**c**) TGA and DTA curves of P-MoS_2_. (**d**) N_2_ adsorption/desorption isotherm and corresponding pore size distribution of P-MoS_2_ and pristine MoS_2_.

The structural information of the two obtained samples was studied by X-ray diffraction (XRD) analysis (Figure 3a). Compared with the highly crystalline pristine MoS_2_ in the 2H-MoS_2_ phase (JCPDS card number 37–1492), all peaks of the P-MoS_2_ are broadened due to nanoscale effects and crystal structural defects. The weakening of the (002) diffraction peak of P-MoS_2_ indicates a low stacking height along this direction [34,35]. Meanwhile, the (002) peak of P-MoS_2_ slightly shifts to a lower angle (14.2° → 13.96°), indicating the formation of sulfur vacancies in MoS_2_ [36,37]. In addition, a new peak appeared at 9.8°, which is related to the presence of P, leading to the formation of a stacking layer, thus extending the c-axis of MoS_2_. According to Bragg’s law, the lattice spacing of the stacking layer at 9.8° is consistent with the spacing in TEM. Due to P’s larger atomic radius than S, P atoms were introduced into the MoS_2_ matrix, causing the lattice to expand and contributing to the widening of interlayer spacing [25,38,39]. Figure 3b exhibits the Raman spectra of P-MoS_2_. Three typical Raman scattering peaks at 280 cm^−1^ (E^1^_g_), 234 (J_2_) cm^−1^, and 334 cm^−1^ (J_3_) are attributed to the octahedral coordination of metal 1T-MoS_2_ [40,41,42]. P-MoS_2_ exhibits two typical characteristic peaks at 376 cm^−1^ and 394 cm^−1^, corresponding to the in-plane (E^1^_2g_) and out-of-plane (A^1^_g_) modes of 2H MoS_2_, which are clearly distinct from pristine MoS_2_. The distance between the E^1^_2g_ and A^1^_g_ peaks is 18 cm^−1^ for P-MoS_2_ and 23 cm^−1^ for bulk MoS_2_. Thus, the moving peak of A^1^_g_ might represent the substantial out-of-plane vibration, indicating that the decreased van der Waals force, together with the enlarged interlayer spacing of MoS_2_ caused by P-embedding, is favourable for Zn^2+^ movement and storage [5,43]. The weaker intensity of the E^1^_2g_ peak in P-MoS_2_ is weaker compared to other samples, thereby proving the presence of Sv in the crystal structure [44].

The TGA-DTA results for the P-MoS_2_ shown in Figure 3c show the initial mass loss of 11.05 wt% at 135 °C due to surface-adsorbed water evaporation. The mass loss of 12.01 wt% between 135 and 350 °C corresponds to the water loss integrated into the crystal structure, implying that the enlarged layer spacing of P-MoS_2_ is probably caused by water insertion [45]. The additional weight loss of 9.69% over 350 °C may be caused by O atoms, which are utilized to aid P atoms embedded in the MoS_2_ structure, with the unsaturated S atoms resulting In the reaction 2MoS_2_ + 7O_2_ → 2MoO_3_ + 4SO_2_ [46,47]. The first derivative curve of the related DTA curve shows a visible endothermic peak at 350 °C. This peak is caused by the transformation of the crystal structure of MoS_2_, which is confirmed by XRD analysis. Moreover, the cumulative pore volume and BET surface area of P-MoS_2_ and pristine MoS_2_ were also measured. As shown in Figure 3d, P-MoS_2_ and pristine MoS_2_ exhibited typical type IV isotherms with hysteresis loops, indicating mesopores between MoS_2_ nanosheets. Nevertheless, the hysteresis of P-MoS_2_ starts at a lower pressure region (P/P_0_ ≈ 0.5) than that of pristine MoS_2_ (P/P_0_ ≈ 1), showing their different porous nature. Furthermore, the wide hysteresis of P-MoS_2_ indicates its increase in porosity. According to the pore volume–pore size distribution curve, P-MoS_2_ has more abundant mesoporous structures, which is conducive to electron transport. The increased specific surface area of P-MoS_2_ is 51.572 m^2^ g^−1^, which is significantly more than pristine MoS_2_ (11 m^2^ g^−1^). The increased specific surface area improves the interaction between the material’s exposed active sites and the electrolyte, which favors increasing the number of active sites for Zn^2+^ storage. These results clearly show that the P-embedding strategy can greatly increase the surface area and pore volume of MoS_2_. To evaluate the hydrophilicity of P-MoS_2_, we performed a contact angle test. Water contact angles of P-MoS_2_ drop from 54.46° to 38.8° (Figure S6), suggesting improved hydrophilicity and beneficial to the diffusion of Zn^2+^ [6].

**Figure 4:**
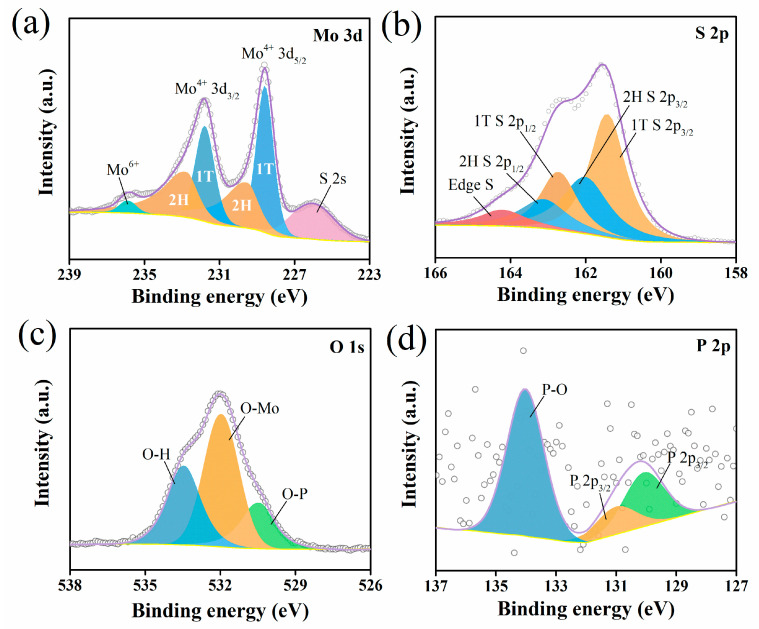
XPS spectra of P-MoS_2_: (**a**) Mo 3d, (**b**) S 2p, (**c**) O 1s, and (**d**) P 2p.

X-ray photoelectron spectroscopy (XPS) reveals detailed valence states and chemical contents of manufactured materials. The survey XPS spectra for P-MoS_2_ and is displayed in Figure S7. As shown in Figure 4a, the XPS spectra of the Mo 3d scan contain two sets of doublet peaks (228.55 and 231.76 eV; 229.5 and 232.75 eV), which belong to Mo 3d_5/2_ and Mo 3d_3/2_ of Mo^4+^ for 1T-MoS_2_ and 2H-MoS_2_ [19,48,49]. This suggests the coexistence of 2H and 1T in P-MoS_2_, which is compatible with Raman data. Similarly, differences can be observed in the S 2p spectra in Figure 4b, where peaks at 161.4 (S 3d_3/2_ of S^2−^) and 162.7 eV (S 3d_1/2_ of S^2−^) attributed to 1T MoS_2_, while peaks at 162.0 and 163.1 eV ascribed to 2H-MoS_2_ [49]. After fitting S 2p spectrum, an extra peak can be seen at 164.19 eV, according to the relevant literature, ascribed to the edge S [50]. Furthermore, using deconvolutions of Mo 3d, the proportion of 1T is estimated at 53% (Table S1), which is equivalent to the product obtained from chemical exfoliation [21]. Meanwhile, the existence of the O 1s signal offers conclusive proof of oxygen-assisted P-embedding in P-MoS_2_ (Figure 4c). It can be deconvoluted into three peaks at 530.42, 531.94, and 533.44 eV, which is ascribed to the P-O bond, Mo-O bond, and adsorbed water, respectively. The analysis shows that oxygen plays an auxiliary role in P-embedding and can also make P-MoS_2_ form a three-dimensional beautiful marigold structure in a bonding manner. P 2p signals were detected at 134.02, 130.99, and 130.06 eV (Figure 4d), confirming the existence of P. The dominant P signal at 134.6 eV can be allocated to the PO_4_^3−^ species, whereas the remaining two should be attributed to Mo-P, indicating that P atoms are embedded in the MoS_2_ lattice [44,51]. Furthermore, elemental composition analysis reveals that the Mo/S ratio is around 1:1.73 (Table S2), which is much lower than the 1:1.95 for MoS_2_ by XPS. This finding implies that P-MoS_2_ has a substantial number of S vacancies [5,20,45].

### 3.2. Electrochemical Performance of Aqueous Zn-Ion Batteries and Kinetic

**Figure 5:**
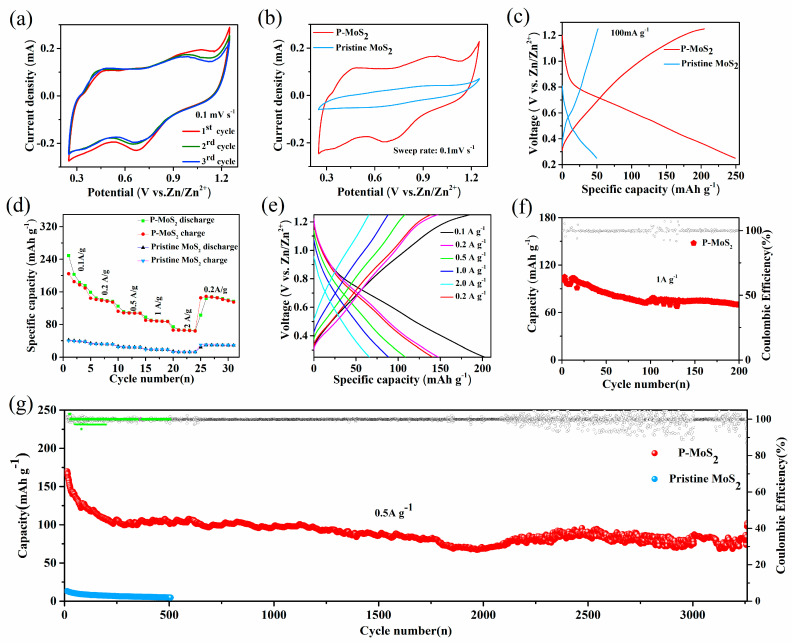
Electrochemical performance of P-MoS_2_ and pristine MoS_2_ (**a**) CV curves at 0.1 mV s^−1^. (**b**) CV at 0.1 mV s^−1^ rate of P-MoS_2_ and Pristine MoS_2._ (**c**) Discharge/charge curves at 0.1 A g^−1^. (**d**) Rate performance. (**e**) First cycle capacity at different cycles. (**f**) Two hundred cycling stability at 1 A g^−1^. (**g**) Long-term cycling stability at 0.5 A g^−1^.

To investigate the Zn^2+^ storage capacity of the prepared samples as stand-alone cathodes for AZIBs, CR2016 coin cells were manufactured in the air environment (see the experimental section for details). Cyclic voltammetry (CV) curves in a voltage window of 0.25 to 1.25 V of 0.1 mV s^−1^ are shown in Figure 5a,b. In contrast to pristine MoS_2_, P-MoS_2_ has two redox peaks: the cathodic peak at 0.65 V linked to Zn^2+^ insertion (possibly overlapping or merging with the reduction in Mo^6+^/Mo^4+^) and an anodic peak at 0.98 V is associated to Zn^2+^ inlay removal [23]. Furthermore, the second and third CV cycles of the two electrodes practically coincide, showing high reversibility. Surprisingly, the peak area of P-MoS_2_ is considerably larger than that of pristine MoS_2_, showing that the enlarged layer spacing and Sv can significantly boost volume capacity. The P-MoS_2_ charge/discharge (CD) curves are comparable with the CV data (Figure 5c), which shows an intercalation plateau of P-MoS_2_ approximately 0.6 V. In addition, the specific capacity of P-MoS_2_ is 249 mA h g^−1^, which is more than five times that of pristine MoS_2_ (50 mA h g^−1^), significantly better than the well-known Zn^2+^ intercalation host, i.e., the Chevrel Phase Mo_6_S_8_ (60 mA h g^−1^ at 0.06 A g^−1^) [52,53]. Meanwhile, the capacity of several modified MoS_2_ materials is compared at 0.1 A g^−1^ in Table S3. Figure 5d shows display ratio capability with the volumes of P-MoS_2_, which are 249, 158, 125, 97, and 75 mAh g^−1^ at 0.1, 0.2, 0.5, 1, and 2 A g^−1^, respectively, and Figure 5e shows the charge/discharge curves of the first cycle capacity. P-MoS_2_ capacitance achieves 143 mAh g^−1^ (91% capacity retention) when it reaches 0.2 A g^−1^. These hint at the excellent rate characteristic and rapid dynamics of P-MoS_2_. In contrast, pristine MoS_2_ offers rather low capacities (0–45 mAh g^−1^) at a variety of current densities. P-MoS_2_ demonstrates a consistent capacity of 70 mAh g^−1^ after 200 cycles at 1 A g^−1^ to further illustrate its outstanding working life (Figure 5f). The stability was further investigated at 0.5 A g^−1^ in Figure 5g.

P-MoS_2_ maintains a capacity of 102 mAh g^−1^ after 3260 cycles, achieving capacity retention of 70%, with the efficiency of the electrode being close to 100%. However, the cycling stability of the P-MoS_2_ electrode seems rather poor after 2000 cycles, which is attributed to the irreversible structural damage, volume changes, and unstable 1T phase during the continuous charging and discharging process [54,55]. In comparison, pristine MoS_2_ has almost zero capacity, which is related to its poor conductivity and unstable structure [16,23].

**Figure 6:**
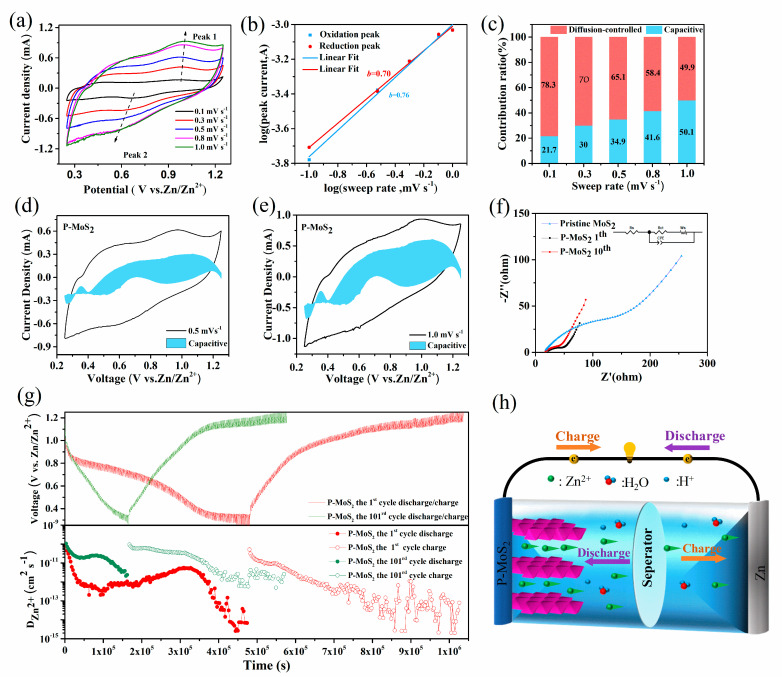
Electrochemical kinetics characterization for the P-MoS_2_ electrode. (**a**) CV curves at various scan rates. (**b**) Log (*i*) versus log (*v*) plots of the redox peaks corresponding to the CV data. (**c**) Capacitive separation curves at 0.5 mV s^−1^ (**d**) Capacitive separation curves at 0.5 mV s^−1^. (**e**) Capacitive separation curves at 1 mV s^−1^. (**f**) EIS spectra, insert image is the fitted equivalent circuit models. (**g**) Discharge/charge GITT profiles and corresponding D_Zn_. (**h**) Schematic diagram for Zn-storage mechanism of P-MoS_2_.

The results show that P-MoS_2_ outperforms pristine MoS_2_ in terms of reversible capacity, cycle stability, and rate capacity. To better understand the electrochemical reaction behavior of the P-MoS_2_ electrode, CV curves at varied scan speeds (0.1–1.0 mV s^−1^) are tested (Figure 6a and Figure S8). The oxidation peak gradually shifted toward high potential with an increasing scan rate, and the reduction peak shifted toward low potential because the electrode polarization broadened at a high scan rate. Whether pseudocapacitive behavior is present or absent depends on the kinetic equation of the reaction:

(1) $$i=av^{b}$$

In between, *i* is the peak current, the unit is A, *v* is the sweep speed, the unit is mV/s, *a* and *b* are the adjustment parameters, and *b* is the slope value of the log (*i*) vs. log (*b*) graph. In particular, *b* = 0.5 indicates that the discharge-specific capacity belongs to the diffusion process control, and *b* = 1 implies the pseudocapacitance control. When *b* is between 0.5–1, there is both diffusion and pseudocapacitance behavior [56]. Obviously, Figure 6b indicates the *b*-values of peak 1 and peak 2, which are 0.76 and 0.70, showing that the redox reaction of the P-MoS_2_ electrode consists of diffusion and capacitive processes. Additionally, the capacitive contribution and diffusion contributions of P-MoS_2_ may be evaluated based on the equation proposed by Dunn [57,58]:

(2) $$i(V)=k_{1}v+k_{2}v^{1/2}$$

(3) $$i(V)/v^{1/2}=k_{1}v^{1/2}$$

At a certain voltage (*v*), *i* is the total current response, where $k_{2}v^{1/2}$ and $k_{1}$*v* are diffusion-controlled and capacitance-dominated contributions. Figure 6c–e reveals the respective contribution rates at different scan rates. Notably, the capacitive contribution increases from 21.7% at 0.1 mV s^−1^ to 50.1% at 1 mV s^−1^. The pseudocapacitance effect is slowly dominant, indicating that the surface reaction is faster than the internal diffusion reaction, which accelerates the intercalation and extraction of Zn^2+^ in the material. Moreover, the diffusion characteristics of P-MoS_2_ are more obvious, which can be attributed to the marigold-shaped structure and wide layer spacing [59]. The impedance of P-MoS_2_ and pristine MoS_2_ is evaluated by EIS, as shown in Figure 6f. The magnitude of the charge transfer resistance is reflected in the semicircle radius of the high-frequency zone (Rct, Table S4). Clearly, results show that the charge transfer impedance of P-MoS_2_ is measured at 42.82 Ω, which is lower than the pristine MoS_2_ under the same conditions. In addition, Rcts of pristine MoS_2_ are markedly over 180 Ω, indicating that expanded layer spacing and S vacancies improve charge-transfer dynamics. Because of the activation of the active material, the charge transfer impedance of P-MoS_2_ was lowered during the first ten cycles.

To examine the solid-state diffusion kinetics of Zn^2+^ intercalation in P-MoS_2_, we investigated GITT, which has been widely used to assess ionic diffusivity, offering insight into electrode kinetics. Figure 6g shows the GITT of P-MoS_2_ before and after 100 cycles, respectively; obviously the P-MoS_2_ cell mainly provides a low Zn^2+^ diffusion coefficient of around 10^−15^–10^−10^ cm^2^ s^−1^ at the 1st cycle. After 100 cycles, the diffusion coefficient stability was increased to 10^−12^–10^−10^ cm^2^ s^−1^, which is attributed to the activation of the electrode and the opening of ion channels during the numerous discharging/charging progress [60,61]. The above electrochemical analysis shows that the Zn^2+^ insertion kinetics of P-MoS_2_ is accelerated, leading to a higher specific capacity. When the P atoms are embedded in the MoS_2_ lattice, the expanded layer spacing of MoS_2_ allows for easy Zn^2+^ desertion. In addition, the P-embedding induces 1T-MoS_2_ and abundant sulfur vacancies.

The material has excellent electrical conductivity and facilitates electron transfer. In addition, P-MoS_2_ possesses a large amount of interlayer water and substantially increased hydrophilicity. As demonstrated in previous studies, interlayer water can act as an electrostatic shield, weakening the interaction of Zn^2+^ with the host material’s framework, lowering the diffusion energy barrier, and accelerating the migration efficiency of Zn^2+^ [62,63]. Benefiting from these synergistic effects, P-MoS_2_ exhibits satisfactory performance. On the basis of the aforementioned tests and analysis, the electrochemical mechanism of P-MoS_2_ proposed in Figure 6h is shown, and the various electrochemical reactions that may occur with P-MoS_2_ and Zn are classified as the following:

(4) $$Cathode:x{Zn}^{2+}+2xe^{-}+P-MoS_{2}↔{Zn}_{x}P-MoS_{2}$$

(5) $$Anode:x{Zn}^{2+}+2xe^{-}↔xZn$$

where *x* is the Zn^2+^ content per unit of P-MoS_2_ in the insertion state. Figure S9 shows that the Faraday equation may calculate 0.65 Zn^2+^ per unit based on the discharge curve at 100 mA g^−1^. Furthermore, 0.65 Zn^2+^ per unit was removed from the P-MoS_2_ during the charging procedure.

## 4. Conclusions

In summary, marigold-shaped 1T-MoS_2_ material with rich S vacancy and expanded interlayer spacing to 0.98 nm was fabricated via the oxygen-assisted method, which found the 1T phase content (53%) and extensively analyzed their performance as cathode materials for ZIBs. Meanwhile, the electrochemical investigation revealed that the S vacancy and interlayer spacing generated by P-embedding are particularly advantageous to the rapid diffusion of Zn^2+^ in P-MoS_2_ and boost the Zn^2+^ storage capacity of the marigold-shaped nanosheets. In comparison to the pure MoS_2_ equivalent, P-MoS_2_ nanosheets have a great specific capacity and display outstanding continuous cycle capabilities in ZIBs. When the current density is 0.1 A g^−1^, the discharge capacity can reach 249 mAh g^−1^. In particular, it displayed a high specific discharge capacity of 105 mAh g^−1^ with a capacity retention of 70% after 3260 cycles at the current density of 0.5 A g^−1^ and an excellent capacity retention of 70 mAh g^−1^ at 1 A g^−1^. This work offers new ideas for designing MoS_2_ as cathode materials for ZIBs.

## Data Availability

Not applicable.

## Supplementary Materials

The following supporting information can be downloaded at: https://www.mdpi.com/article/10.3390/nano13071185/s1. Figure S1. SEM images of the sample of Pristine MoS2; Figure S2. TThe corresponding atomic intensity profile along the dotted red line for P-MoS2; Figure S3. TEM images of (a,b) Pristine MoS2; Figure S4. Line scan of the HRTEM image; Figure S5. EDS spectrum; Figure S6. Contact angles with water for (a) P-MoS2 and (b) Pristine MoS2; Figure S7. XPS spectra of full scan for P-MoS2; Figure S8. The initial five CV curves of (a) Pristine MoS2 and (b) CV curves at various scan rates of Pristine MoS2; Figure S9. Inital charge-discharge profile of P-MoS2 nanosheets at 0.1A g-1; Table S1. Phase content of Mo 3d in each sample; Table S2. Atomic percentages of P-MoS2 by XPS measurement; Table S3. Comparisons of performance of MoS2 synthesized under different conditions in neutral media; Table S4. Charge transfer resistance of MoS2 samples. References [64,65] are cited in Supplementary Materials.
