# Supplementary material for: Enlarged Interlayer Spacing of Marigold-Shaped 1T-MoS2 with Sulfur Vacancies via Oxygen-Assisted Phosphorus Embedding for Rechargeable Zinc-Ion Batteries
Source: Nanomaterials (Basel). 2023 Mar 27;13(7):1185. doi: 10.3390/nano13071185 (PMC10096869; doi:10.3390/nano13071185)
Supplement: Supplementary file 1 [file nanomaterials-13-01185-s001.zip › nanomaterials-2277081-supplementary.pdf]

Supporting Information

Article

Enlarged Interlayer Spacing of Marigold-Shaped 1T-MoS_2 with Sulfur Vacancies via Oxygen-Assisted Phosphorus Embedding for Rechargeable Zinc-Ion Batteries

Qinhu Xu, Xinyu Li *, Luchen Wu, Zhen Zhang, Yong Chen, Ling Liu and Yong Cheng *

College of Science & Ministry-Province Jointly-Constructed Cultivation Base for State Key Laboratory of Processing for Mom-Ferrous Metal and Featured Materials & Key Lab. of Nonferrous Materials and New Processing Technology & Network and Information Center, Guilin University of Technology, Guilin 541004, China

* Correspondence: lixinyu5260@163.com (X.L.); hb_cy@163.com (Y.C.)

Chemicals: Conductive acetylene black, polyvinylidene fluoride (PVDF) were purchased from Guangdong Canrd New Energy Technology Co., Ltd. Ammonium molybdate tetrahydrate ($(NH_4)_6Mo_7O_{24}\cdot4H_2O$) and N-methyl pyrrolidone (NMP) were from Shanghai Aladdin Bio-Chem Technology Co., LTD. Thiourea (CH_4N_2S) and Sodium hypophosphite (NaH_2PO_2) were purchased from Shanghai Macklin Biochemical Co., Ltd. Zinc trifluoromethanesulfonate ($Zn(CF_3SO_3)_2$) was purchased from TCI (Shanghai) Development Co., Ltd.

The electrochemical tests were conducted on the two-electrode CR2016 type coin cells. The active material (P-MoS_2) was dispersed in N-methyl pyrrolidone (NMP) solution with polyvinylidene fluoride (PVDF) and Super P with an 7:2:1 weight ratio. P-MoS_2 /Zn batteries were assembled using P-MoS_2 cathode and zinc plate anode with 3 M $Zn(CF_3SO_3)_2$ aqueous solution as an electrolyte as well as a microporous glass fiber membrane (Whatman) as the separator.Calculation method of surface-controlled capacitive and diffusion-limited behaviors and GITT tests .Cyclic voltammetry (CV) measurements were conducted using a Princeton Analytical electrochemical workstation. The galvanostatic charge/discharge test and the galvanostatic intermittent

titration technique (GITT) of P-MoS_2 /Zn batteries were tested by the Neware battery tester (CT4008). The Zn^{2+} diffusivity (D^{GITT}) can be calculated by the following equation:[64,65]

$$D^{GITT} = \frac{4L^2}{\pi\tau}\left(\frac{\Delta E_S}{\Delta E_\tau}\right)^2 \quad \text{(S1)}$$

Where t and τ represent the duration pulse (s) and relaxation time (s), respectively. L corresponds to the Zn^{2+} diffusion length (equal to the thickness of the electrode). ΔE_s is the steady-state potential change (V) by the current pulse. ΔE_t is the voltage change (V) during the constant current pulse (eliminating the voltage changes after relaxation time).

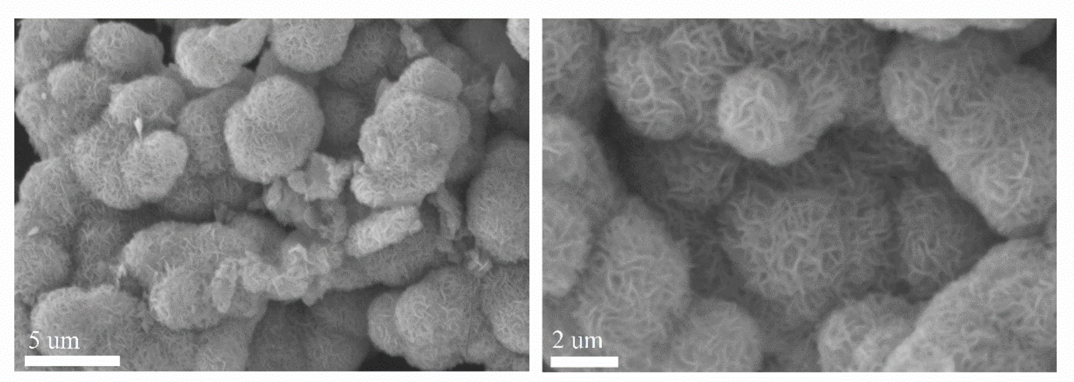

Figure S1. SEM images of the sample of Pristine MoS_2.

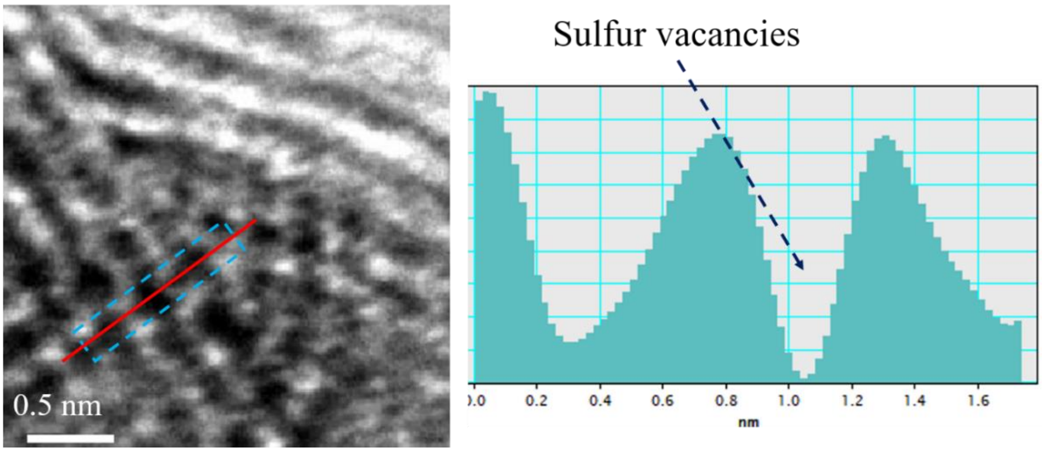

Figure S2. The corresponding atomic intensity profile along the dotted red line for P-MoS_2

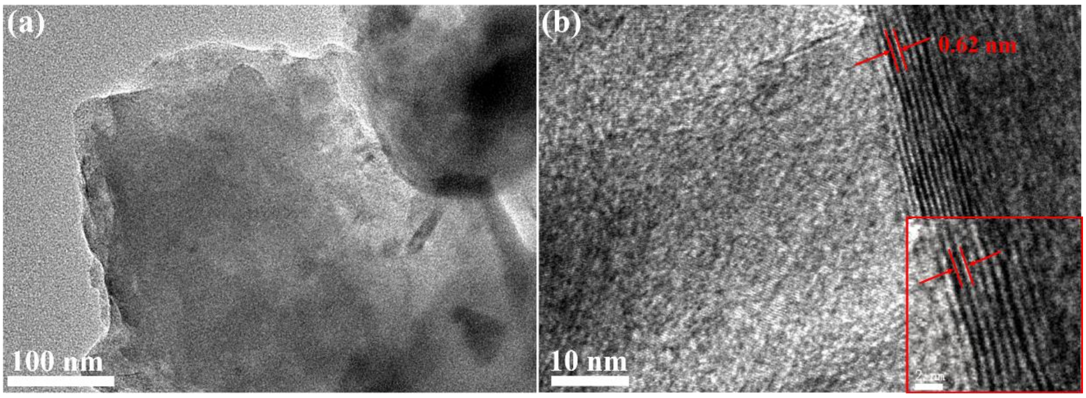

Figure S3. TEM images of (a, b) Pristine MoS_2

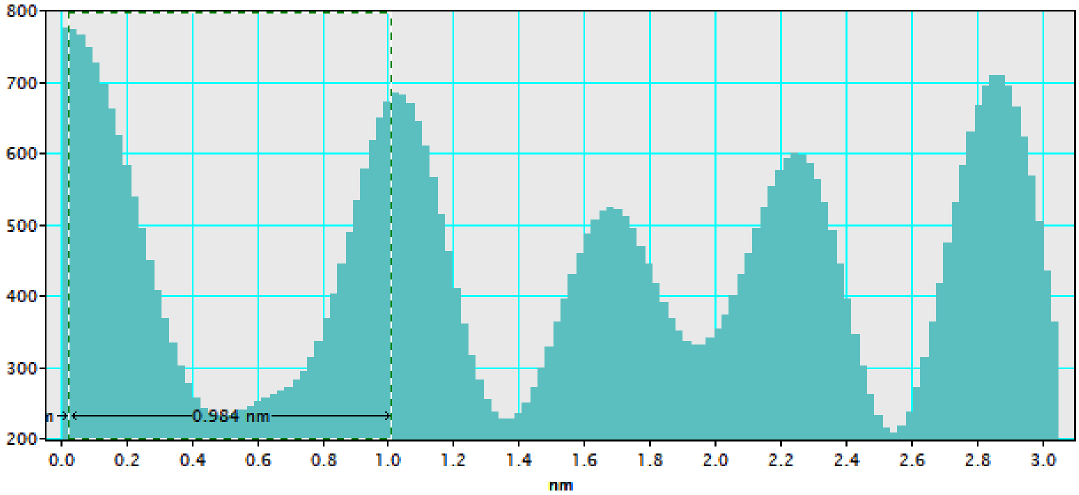

Figure S4. Line scan of the HRTEM image

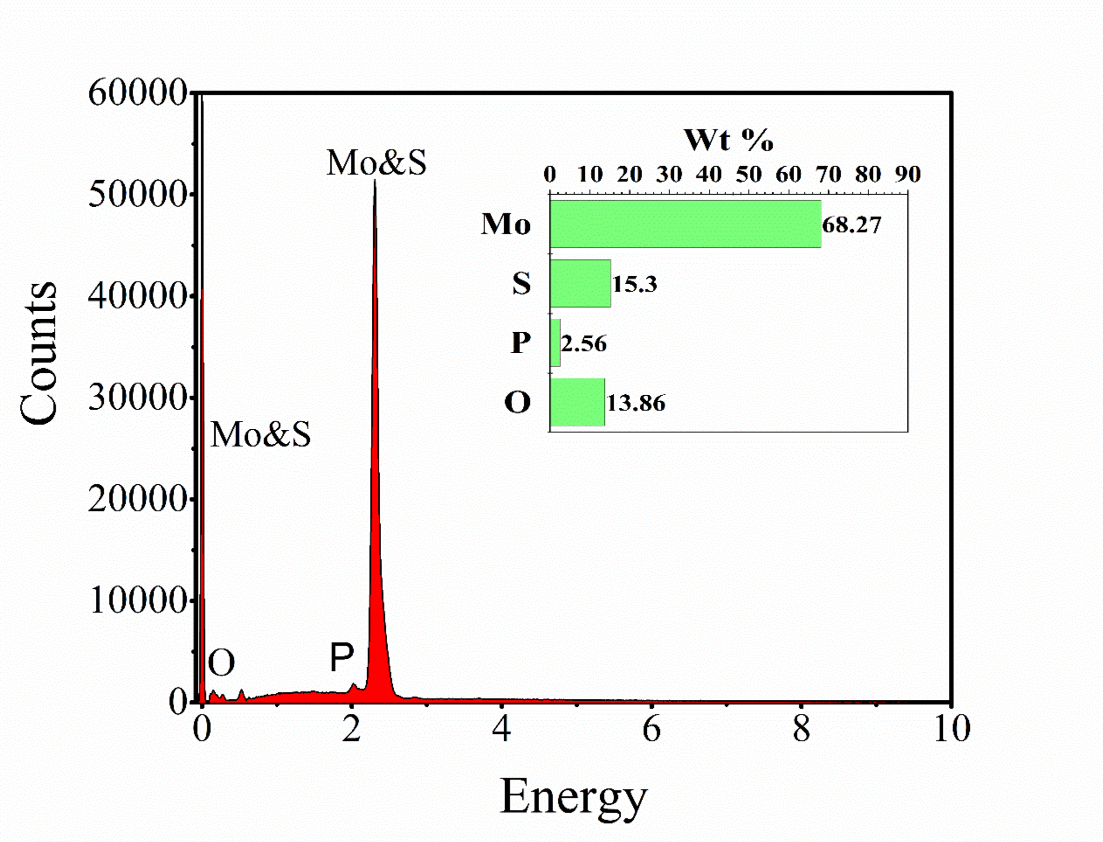

Figure S5. EDS spectrum

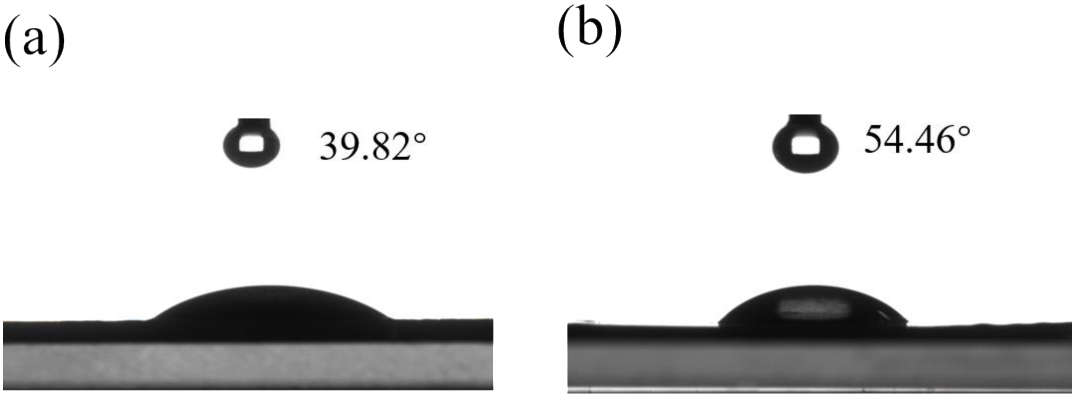

Figure S6. Contact angles with water for (a) P-MoS_2 and (b) Pristine MoS_2.

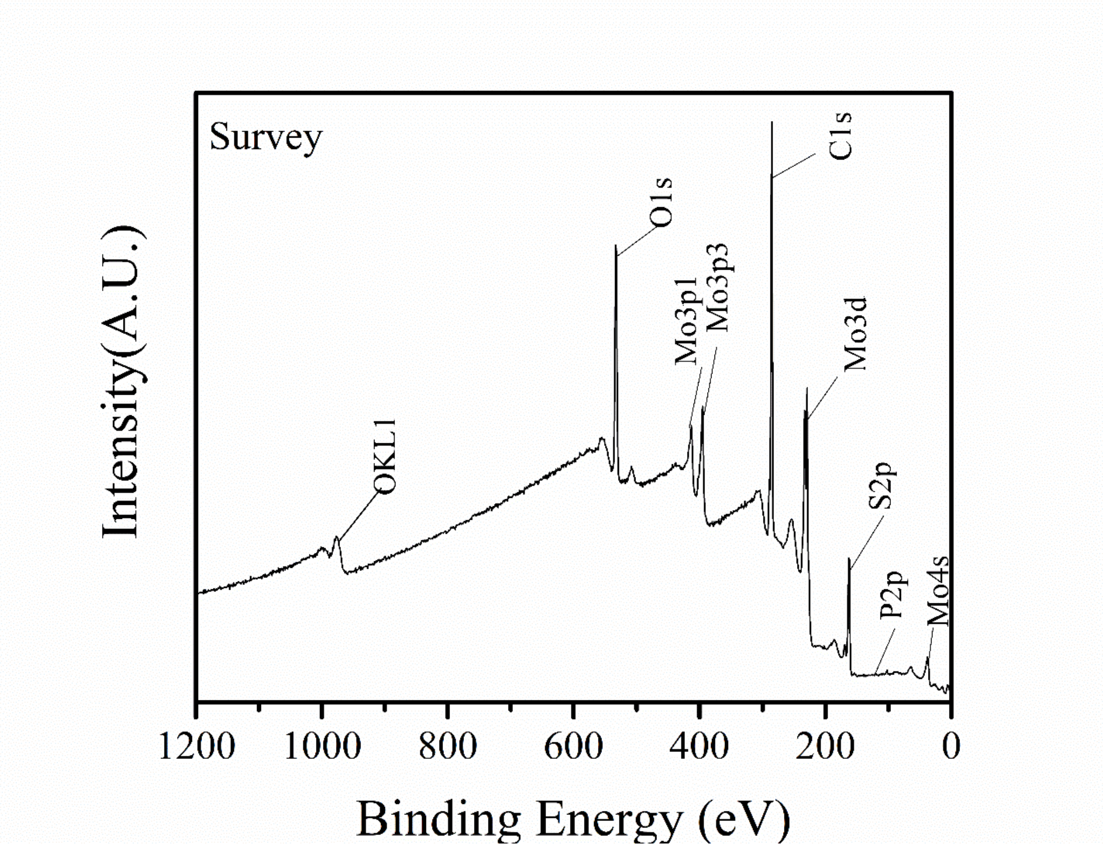

Figure S7. XPS spectra of full scan for P-MoS_2.

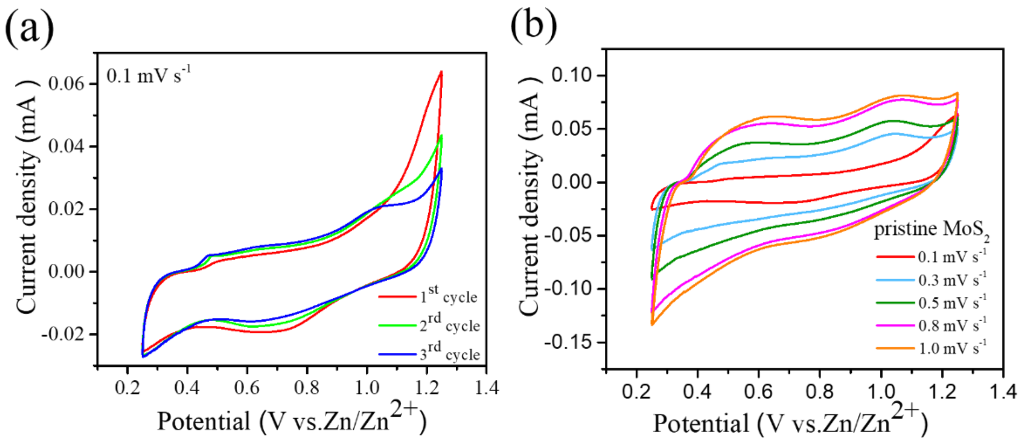

Figure S8. The initial five CV curves of (a)Pristine MoS_2 and (b) CV curves at various scan rates of Pristine MoS_2.

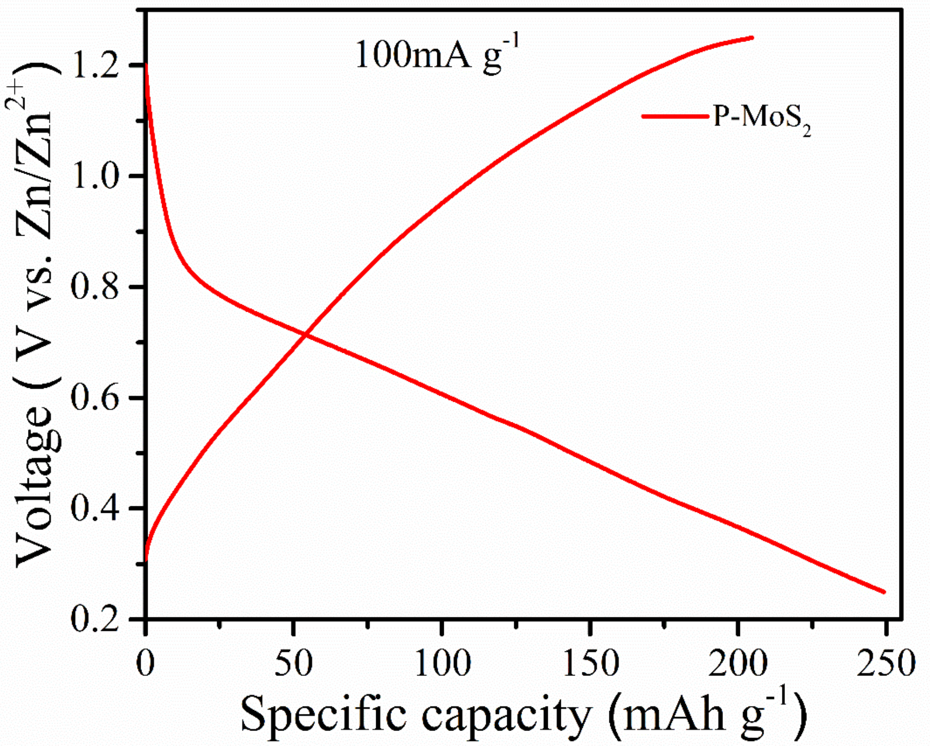

Figure S9. Inital charge-discharge profile of P-MoS2 nanosheets at 0.1A g^{-1}

At the cathode: $P - MoS_2 + xZn^{2+} + 2xe^- = Zn_xP - MoS_2$

At the anode: $xZn^{2+} + 2xe^- = xZn$

n is determined based on the following equations:

$$Q_{theoretical} = \frac{2nF}{3600*M}\ mAh/g \quad \text{(S2)}$$

(F = NA*e = 96500 C/mol, NA = 6.02×10^{23}, 1 Ah = 1 A×3600s = 3600 C, M= 160.07 g/mol)

Table S1. Phase content of Mo 3d in each sample

Phase	Binding Energy(eV)	P-MoS_2
2H	229.50	22.3%
	232.75	22.3%
1T	228.55	30.6%
	231.76	22.4%

Table S2. Atomic percentages of P-MoS_2 by XPS measurement

Atomic(%)	Mo 3d	S 2p	O 1s	P 2p	S/Mo
P-MoS_2	6.29	10.89	18.07	0.61	1.73<1.95

Table S3. Comparisons of performance of MoS_2 synthesized under different conditions in neutral media.

Positive materials (experimental group)	Specific capacity @ 0.1 A g-1 (mAh g^{-1})	Reference
P-MoS_2	249 mAh g^{-1}	This work
Glu-MoS_2	182 mAh g^{-1}	*Chem. Eng. J.* 416(2021)127704
MoS_{2-x}	128 mAh g^{-1}	*Energy Storage Mater.* 2019, 16,527-534
MoS_2-O	232 mAh g^{-1}	*Nano Lett*. 2019, 19, 3199-3206.
$Co_xMo_{1-x}S_2$	305.4 mAh g^{-1}	*Energy Storage Mater*. 55 (2023) 1-11
MoS_2/rGO	283 mAh g^{-1}	*Adv. Mater*. 2021, 33, 2007480

Table S4. Charge transfer resistance of MoS_2 samples.

Samples	Charge transfer resistance (Rct, Ω)
P-MoS_2	42.82
Pristine MoS_2	184.8
P-MoS_2 after 10 cycles	40.58

References

64. Zhang, N.; Cheng, F.; Liu, Y.; Zhao, Q.; Lei, K.; Chen, C.; Liu, X.; Chen, J. Cation-Deficient Spinel ZnMn2O4 Cathode in Zn(CF3SO3)2 Electrolyte for Rechargeable Aqueous Zn-Ion Battery. *Journal of the American Chemical Society* **2016**, *138*, 12894-12901, doi:https://doi.org/10.1021/jacs.6b05958.
65. Shaju, K.M.; Subba Rao, G.V.; Chowdari, B.V.R. Li ion kinetic studies on spinel cathodes, Li(M1/6Mn11/6)O4 (M = Mn, Co, CoAl) by GITT and EIS. *Journal of Materials Chemistry* **2003**, *13*, 106-113, doi:http://dx.doi.org/10.1039/B207407A.
